# Supplementary material for: Exploring Gut Microbiome Variations between Popillia japonica Populations of Azores
Source: Microorganisms. 2023 Jul 31;11(8):1972. doi: 10.3390/microorganisms11081972 (PMC10459852; doi:10.3390/microorganisms11081972)
Supplement: Supplementary file 1 [file microorganisms-11-01972-s001.zip › microorganisms-2521191-supplementary.pdf]

microorganisms

MDPI

Supplementary Materials

Exploring Gut Microbiome Variations between *Popillia japonica* Populations of Azores

Jorge Frias [1,*], Anna Garriga [1,2], Ángel Peñalver [1], Mário Teixeira [1], Rubén Beltrí [1], Duarte Toubarro [1] and Nelson Simões [1,*]

[1] Centro de Biotecnologia dos Açores, Faculdade de Ciências e Tecnologia, Universidade dos Açores, 9500-321 Ponta Delgada, Portugal

[2] Departament de Biologia Animal, Vegetal i Ecologia, Facultat de Biociències, Universitat Autònoma de Barcelona, 08193 Bellaterra, Spain

* Correspondence: jorge.mv.frias@uac.pt (J.F.); nelson.jo.simoes@uac.pt (N.S.)

1 Supplementary Figures and Tables

1.1 Supplementary Figures

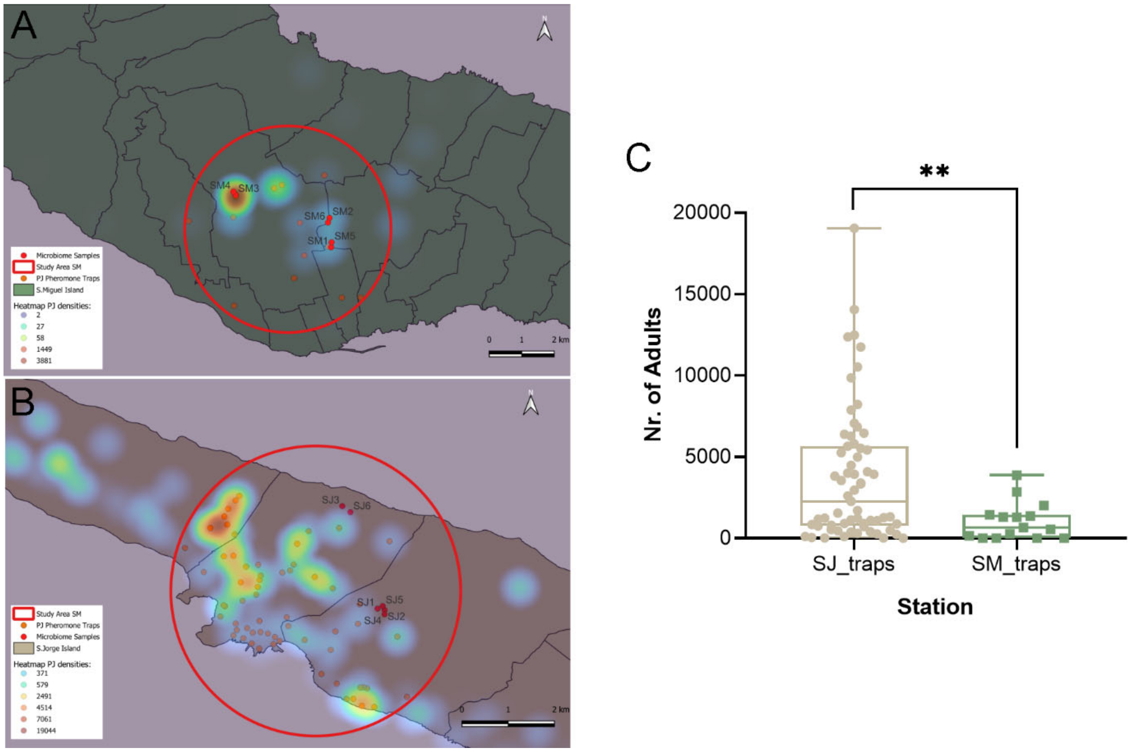

Supplementary Figure S1. Heatmap representing the most affected areas in the two selected islands, São Miguel (low-density site) and São Jorge (high-density site). Study area delineation (red circles 40 Km2) on A) São Miguel and B) São Jorge islands. C) *Popillia japonica* adult trap captures during the year 2021 within their respective study areas. The Mann-Whitney test showed a significant difference between the two study areas ($p = 0.0058$). The double asterisk (**) indicates a significant level of $p < 0.01$.

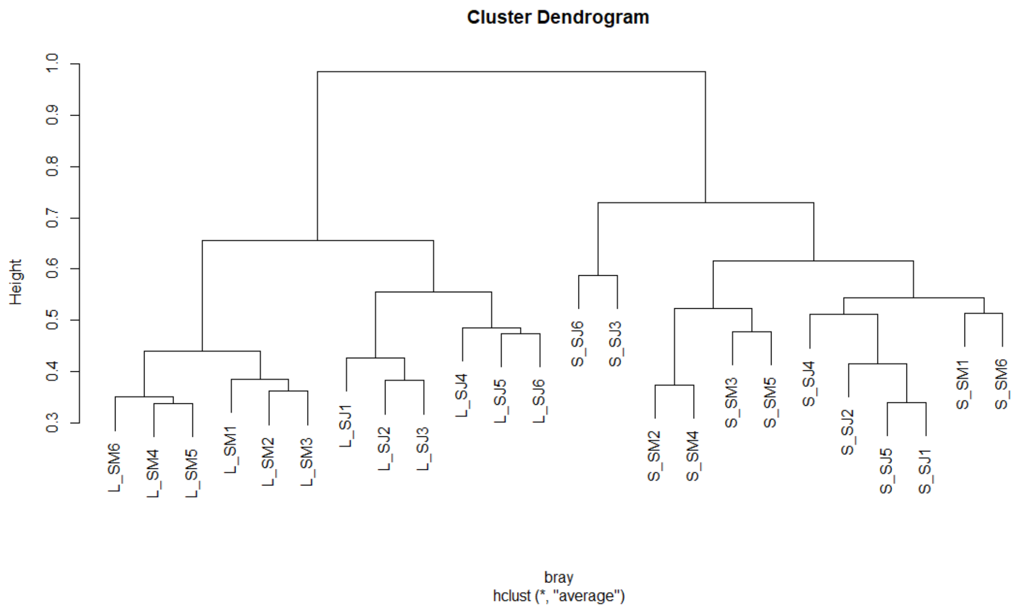

Supplementary Figure S2. Cluster dendogram showing taxa structural composition of soil and larvae microbiomes sampled in high- and low- density sites.

1.2 *Supplementary Tables*

Supplementary Table S1. Sampling metadata for larvae and soil samples collected from high- and low- density sites.

ID sample	Type	Location	Density	Collection	Coordinates
L_SJ1	Larva	São Jorge	High density	19/05/2021	38°41'19.1"N | 28°10'45.4"W
L_SJ2	Larva	São Jorge	High density	19/05/2021	38°41'17.8"N | 28°10'39.1"W
L_SJ3	Larva	São Jorge	High density	19/05/2021	38°42'50.6"N | 28°11'16.9"W
L_SJ4	Larva	São Jorge	High density	19/05/2021	38°41'15.6"N | 28°10'37.2"W
L_SJ5	Larva	São Jorge	High density	19/05/2021	38°41'20.4"N | 28°10'44.1"W
L_SJ6	Larva	São Jorge	High density	19/05/2021	38°42'45.3"N | 28°11'11.7"W
L_SM1	Larva	São Miguel	Low density	19/04/2021	37°46'14.8"N | 25°40'30.4"W
L_SM2	Larva	São Miguel	Low density	19/04/2021	37°46'51.6"N | 25°40'32.5"W
L_SM3	Larva	São Miguel	Low density	19/04/2021	37°47'20.6"N | 25°42'30.7"W
L_SM4	Larva	São Miguel	Low density	19/04/2021	37°47'24.5"N | 25°42'33.1"W
L_SM5	Larva	São Miguel	Low density	19/04/2021	37°46'17.2"N | 25°40'29.2"W
L_SM6	Larva	São Miguel	Low density	19/04/2021	37°46'48.4"N | 25°40'33.4"W
S_SJ1	Soil	São Jorge	High density	19/05/2021	38°41'19.1"N | 28°10'45.4"W
S_SJ2	Soil	São Jorge	High density	19/05/2021	38°41'17.8"N | 28°10'39.1"W
S_SJ3	Soil	São Jorge	High density	19/05/2021	38°42'50.6"N | 28°11'16.9"W
S_SJ4	Soil	São Jorge	High density	19/05/2021	38°41'15.6"N | 28°10'37.2"W
S_SJ5	Soil	São Jorge	High density	19/05/2021	38°41'20.4"N | 28°10'44.1"W
S_SJ6	Soil	São Jorge	High density	19/05/2021	38°42'45.3"N | 28°11'11.7"W
S_SM1	Soil	São Miguel	Low density	19/04/2021	37°46'14.8"N | 25°40'30.4"W
S_SM2	Soil	São Miguel	Low density	12/03/2021	37°46'51.6"N | 25°40'32.5"W
S_SM3	Soil	São Miguel	Low density	19/04/2021	37°47'20.6"N | 25°42'30.7"W
S_SM4	Soil	São Miguel	Low density	06/05/2021	37°47'24.5"N | 25°42'33.1"W
S_SM5	Soil	São Miguel	Low density	19/04/2021	37°46'17.2"N | 25°40'29.2"W
S_SM6	Soil	São Miguel	Low density	19/04/2021	37°46'48.4"N | 25°40'33.4"W

Supplementary Table S2. Denoising step from Qiime2 software for all the samples.

Scheme .	sample-type	site	input	filtered	input passed filter (%)	denoised	merged	input merged (%)	non-chimeric	input non-chimeric (%)
L-SJ1	Larva gut	High density	62638	39509	63.08	34146	28842	46.05	28373	45.3
L-SJ2	Larva gut	High density	85174	50729	59.56	44792	39353	46.2	38809	45.56
L-SJ3	Larva gut	High density	84898	51679	60.87	44829	38478	45.32	37335	43.98
L-SJ4	Larva gut	High density	167344	52116	31.14	42547	30991	18.52	29578	17.67
L-SJ5	Larva gut	High density	123204	39521	32.08	31285	21204	17.21	20444	16.59
L-SJ6	Larva gut	High density	118775	36888	31.06	30248	21380	18	20709	17.44
L-SM1	Larva gut	Low density	116057	72185	62.2	65353	58007	49.98	56604	48.77
L-SM2	Larva gut	Low density	75209	47436	63.07	42830	37940	50.45	36884	49.04
L-SM3	Larva gut	Low density	70543	43354	61.46	38738	34142	48.4	33286	47.19
L-SM4	Larva gut	Low density	124702	39366	31.57	32886	25449	20.41	24110	19.33
L-SM5	Larva gut	Low density	139825	44844	32.07	40360	32609	23.32	31969	22.86
L-SM6	Larva gut	Low density	108008	31792	29.43	28069	22702	21.02	22227	20.58
S-SJ1	Soil	High density	69393	39519	56.95	33618	23734	34.2	23226	33.47
S-SJ2	Soil	High density	58307	34242	58.73	28862	20326	34.86	19929	34.18
S-SJ3	Soil	High density	59246	35586	60.06	31912	25573	43.16	24704	41.7
S-SJ4	Soil	High density	150679	36219	24.04	28412	16915	11.23	16076	10.67
S-SJ5	Soil	High density	138046	34727	25.16	28552	17484	12.67	16760	12.14
S-SJ6	Soil	High density	118663	28044	23.63	23838	17075	14.39	15613	13.16
S-SM1	Soil	Low density	56807	32413	57.06	27781	19951	35.12	19454	34.25
S-SM2	Soil	Low density	66558	38356	57.63	32634	22080	33.17	21368	32.1
S-SM3	Soil	Low density	69324	40986	59.12	34471	22283	32.14	21549	31.08
S-SM4	Soil	Low density	132456	30885	23.32	23333	12430	9.38	11910	8.99
S-SM5	Soil	Low density	116573	27865	23.9	22483	13345	11.45	12380	10.62
S-SM6	Soil	Low density	132702	33078	24.93	26655	15737	11.86	15298	11.53
	Total		2445131	961339		818634	618030		598595	

Supplementary Table S3. Simpson index and richness values for all samples.

	Simpson	S.obs	S.chao1	se.chao1	S.ACE	se.ACE
L_SJ1	0.9871	774	774	0.2498384	774.142941	12.9032089
L_SJ2	0.9891	834	834	0	834	13.33434
L_SJ3	0.9926	840	840	0	840	13.18928
L_SJ4	0.9882	638	638	0	638	9.994983
L_SJ5	0.9939	543	543	0	543	9.459617
L_SJ6	0.9901	532	532	0	532	9.079846
L_SM1	0.991	859	859	0	859	12.25505
L_SM2	0.9864	745	745	0	745	12.26245
L_SM3	0.9852	681	681	0	681	12.05165
L_SM4	0.9852	443	443	0	443	9.008649
L_SM5	0.9876	559	559	0	559	9.131714
L_SM6	0.9859	467	467	0	467	8.991073
S_SJ1	0.9957	710	711.428571	1.935033	710.730198	11.479282
S_SJ2	0.9958	601	601.25	0.7367539	601.284593	10.4658814
S_SJ3	0.9915	401	401.333333	0.9260305	401.307692	7.8153126
S_SJ4	0.9909	373	373	0.1248323	373.139165	7.5224649
S_SJ5	0.9941	463	463	0	463	8.253542
S_SJ6	0.972	181	181	0.2493084	181.178642	4.8381721
S_SM1	0.9961	597	597	0.2497905	597.141885	10.4909228
S_SM2	0.9953	605	605.25	0.7367546	605.272727	10.5267941
S_SM3	0.9941	450	450.333333	0.9260594	450.286541	8.7606002
S_SM4	0.9928	368	368	0	368	7.982998
S_SM5	0.9885	268	268	0	268	6.119153
S_SM6	0.992	398	398	0	398	7.91919

Supplementary Table S4. Soil parameters obtained from the soil samples collected: pH, OM (organic matter), P, K, Ca, Mg, silt, sand, clay, and class. In addition, with the climatic conditions from both study sites; pluviometry, temperature, relative humidity, radiation, and wind.

Sample	pH	OM	P	K	Ca	Mg	Silt	Sand	Clay	Class	Pluvi	Temp	Humi	Rad	Wind
S_SJ1	5.8	7.4	28	226	721	184	4	93	3	Sandy	4.77	16.24	79.6	12554	5.65
S_SJ2	6	7.6	11	36	505	108	3	95	2	Sandy	4.77	16.24	79.6	12554	5.65
S_SJ3	5.8	9.4	16	108	534	131	3	94	2	Sandy	4.77	16.24	79.6	12554	5.65
S_SJ4	6	7.6	11	36	505	108	3	95	2	Sandy	4.77	16.24	79.6	12554	5.65
S_SJ5	5.8	7.4	28	226	721	184	4	93	3	Sandy	4.77	16.24	79.6	12554	5.65
S_SJ6	5.4	17.1	4	14	217	72	3	95	2	Sandy	4.77	16.24	79.6	12554	5.65
S_SM1	5.9	10.7	16	55	1234	292	4	93	3	Sandy	5.56	15.84	77.59	13010	3.65
S_SM2	6.1	13	48	337	1367	509	7	90	3	Sandy-loam	5.56	15.84	77.59	13010	3.65
S_SM3	5.9	10.7	16	55	1234	292	4	93	3	Sandy	5.56	15.84	77.59	13010	3.65
S_SM4	5.9	11.2	22.4	111.4	1260.6	335.4	4.6	92.4	3	Sandy	5.56	15.84	77.59	13010	3.65
S_SM5	5.9	10.7	16	55	1234	292	4	93	3	Sandy	5.56	15.84	77.59	13010	3.65
S_SM6	5.9	10.7	16	55	1234	292	4	93	3	Sandy	5.56	15.84	77.59	13010	3.65
